# Self-awareness predicts fitness to drive among adults referred to occupational therapy evaluation

**DOI:** 10.3389/fresc.2022.1005025

**Published:** 2022-11-16

**Authors:** Meirav Rosenfeld, Yael Goverover, Penina Weiss

**Affiliations:** ^1^ Department of Occupational Therapy, New York University, New York, NY, United States; ^2^ Kessler Foundation, East Hanover, NJ, United States; ^3^ Occupational Therapy Department, Rabin Medical Center, Beilinson Hospital, Petach Tikva, Israel

**Keywords:** driving evaluation, self-awarness, processing speed (PS), occupational therapy, community, older (elderly) drivers

## Abstract

**Background:**

Driving is associated with independence, well-being, quality of life, and an active lifestyle. Driving requires cognitive, motor, and visual skills, including self-awareness and processing speed. This study examines whether driver self-awareness, motor processing speed, and cognitive processing speed can predict fitness to drive among individuals referred to occupational therapy evaluation due to concerns about their driving ability.

**Method:**

In this cross-sectional study, 39 participants were referred to off- and on-road driving evaluation to determine their fitness to drive due to changes in health status, advanced age, license renewal requirement, or prior automobile accidents. A registered occupational therapist (OT) classified 23 of the participants as fit to drive and 16 as unfit to drive. Motor and cognitive processing speed were assessed by the Stationary Perception-Reaction Timer and the Color Trails Test, respectively. Driving self-awareness was assessed by comparing the DI and OT evaluations to the participants' estimation of their own on-road driving performance.

**Results:**

The fit-to-drive participants had a better motor and cognitive processing speed than those unfit-to-drive. The unfit-to-drive group overestimated their driving ability, whereas the fit-to-drive group accurately or almost accurately estimated their driving ability. Driving self-awareness was a significant predictor of participants' fitness to drive.

**Conclusions:**

This study demonstrates the importance of self-awareness for predicting fitness to drive among people at risk for compromised driving skills. Thus, driving self-awareness should be addressed as part of fitness-to-drive evaluations and interventions.

## Introduction

Driving a car is a common and meaningful Instrumental Activity of Daily Living (IADL) for many adults (1, 2). It allows them to engage in various activities, from performing daily mundane tasks such as shopping for groceries and commuting to work, to achieving broader life goals such as engaging in one's community and traveling to new places (3). Driving is strongly associated with independence, an active lifestyle, well-being, and quality of life (4, 5). Therefore, it is not surprising that people desire to continue to be able to drive and to maintain their driver's license for as long as possible, even in light of advancing age or changes in health status (3).

Changes in functional and/or health status may affect various skills associated with driving (e.g., cognitive, motor, and visual). Such changes may be due to a new medical diagnosis such as dementia or acquired brain injury (ABI) (6–8), mental health diagnoses such as schizophrenia (9), or various chronic diseases. The aging process may also affect driving ability, with changes in visual functioning and processing (10, 11) or declining cognitive function (12). In fact, chronic medical conditions and age (>75 years) are associated with decreased driving safety and increased risk of traffic accidents and road deaths (13, 14). Thus, changes in health and age may indicate the necessity to re-evaluate one's ability to drive (6, 7).

In most countries, individuals with a change in health status that might have an impact on their driving ability must receive clearance from a licensing jurisdiction to resume driving (15). One method that helps determine driving fitness is an evaluation by a driver rehabilitation specialist, a role primarily undertaken by occupational therapists (OT). As part of the clearance process to be considered fit to drive, drivers are required to undergo a thorough evaluation of off- and on-road driving abilities (3, 7, 16). The off-road section of the evaluation (in-clinic) assesses vision, cognitive, and motor skills (6, 7). The on-road test assesses actual driving skills and is conducted by driving instructors (DIs) and OTs specialized in driving rehabilitation (6, 7, 17). Both components of the evaluation are crucial to understanding the impact of identified impairments on clients' actual driving performance (17, 18).

Driving self-awareness is not consistently assessed during the fitness to drive assessment. In fact, studies examining self-awareness of driving behavior (i.e., driving self-awareness) in individuals with mild cognitive impairments reported contradictory results (19). Self-awareness is a product of the dynamic relationship between knowledge, beliefs, task demands, and the context of a situation (20–22). Although it is not regularly included in the driving evaluation, an inability to recognize one's own strengths and weaknesses, specifically in the context of driving, can be a crucial detriment to driving ability. For example, previous research emphasizes that reduced driving self-awareness is associated with cognitive impairments (23) and that individuals who are unaware of their limitations tend to choose activities beyond their capabilities and do not recognize when they need help. Thus, overestimation of one's driving abilities, and failure to correct errors may cause dangerous safety events (23).

The current study examines whether driving self-awareness and cognitive and motor processing speed can predict fitness to drive among individuals referred to occupational therapy evaluation with a concern regarding their driving ability. In addition, this study assesses whether driving self-awareness can predict fitness to drive above and beyond motor and cognitive processing speed.

## Methods

### Participants

The current cross-sectional study is based on data collected in a driving rehabilitation center at a major hospital in Israel between 2018 and 2019. The study included 39 participants who were older than 65 years of age and who held a valid driver's license when referred to the clinic to determine their fitness to drive. Referrals to evaluate the clients’ driving abilities were made primarily by physicians, allied health care professionals, or self-referral (self and/or family) due to changes in health status, advanced age, license renewal requirement, or previous involvement in a motor vehicle accident. Participants were excluded if they had expressive difficulties. Initial data retrieval included 45 participants; among them, six participants were excluded due to missing data. The study was approved by the hospital's Helsinki Committee for Human Rights.

### Procedures and data collection

As a part of the driving rehabilitation center's project and based on an established protocol to assess fitness to drive (6), the clients underwent an in-clinic evaluation (i.e., off-road evaluation) and an on-road test. The in-clinic evaluation was performed by a registered OT who was also a driving rehabilitation specialist. The on-road test was conducted by both an OT and a licensed driving instructor (DI). Following the completion of both parts of the evaluation, the OT determined the client's driving fitness status (described below). For the purpose of the present study, data, with no identification details, were retrieved from clients' records.

### Measures

The *On-Road Driving Evaluation* (24) is based on the standard test procedure used by the provincial licensing board in Quebec, Canada. The on-road evaluation is based solely on performance analysis made by the OT and the DI, seated in different positions within the car, who scored the driving performance separately. The actual route follows the recommendations of Korner-Bitensky and colleagues (25) and includes eight intersections, both right and left turns, and navigation according to road signs and according to oral instructions. The route starts in a quiet residential environment and ends up in a roadway requiring a speed of more than 70 km/hr. The route is predetermined and clearly documented. While driving along the route, all participants were given directions in the same format and sequence. Driving a dual-brake control motor vehicle, each participant was rated independently by the OT and the DI on 43 items observable driving behaviors that relate to four themes: (1) control of the vehicle; (2) ability to maneuver the vehicle; (3) specific driving skills such as reaction time and paying attention to road signs; and (4) general driving skills such as decision making, planning, and tolerance. Each item was scored on a 5-point Likert scale, ranging from 1 = fail (significant mistake/error, for example, if the DI had to actively intervene by braking or handling the steering wheel) to 5 = competent and safe driving ability (minimal/no intervention by the DI, just verbal or non-verbal gestures). The total score reflects the amount and quality of intervention required by the DI during on-road evaluation. Note that the scoring does not include any subjective or verbal input by the participant. The on-road driving evaluation is considered the gold standard for driving ability; however, more studies need to be done to further assess its validity and reliability (26).

The *in-clinic, off-road evaluation* included the following assessments: *Color Trails Test* (CTT), *Stationary Perception-Reaction Timer,* and *driving self-awareness questionnaire*.

The *CTT* (27), a paper-and-pencil test of visual scanning processing speed, was designed as a culturally fair analog to the Trail Making Test (TMT), developed to be language free and applicable across cultures. The CTT retains the same psychometric properties as the TMT but relies on the use of the universal concepts of color and numbers instead of the English alphabet letters. It is comprised of two subtests. The first, CTT-1, consists of 25 circled numbers (1–25), with even numbers on a yellow background and odd numbers on a pink background. The respondent is instructed to rapidly connect the circles in consecutive order. The second subtest, CTT-2, consists of double the stimuli as the CTT-1, with two sets of the 25 numbers in each color (pink and yellow). For this study, similar to previous studies (28, 29), only raw completion time of CCT-1 was analyzed, since the time component is considered essential for driving performance, regardless of age. Additionally, the CTT-2 had not been administrated to some participants in the unfit group because of its increased cognitive demands on frontal systems functioning (flexibility, increased information processing) (29, 30). Nonetheless, previous research indicates the validity of CTT-1 as a brief screening test in driving evaluation processes that could identify participants most at risk for unsafe driving behaviors (29). In this current study, we refer to the CTT1 as cognitive processing speed.

*Stationary Perception-Reaction Timer* is a desk-mounted stationary system that includes a personal computer (PC), steering wheel, two pedals (accelerator and brake), and a software that records the clients' reaction time in milliseconds. The score of the Stationary Perception-Reaction Timer is referred to as motor processing speed.

A *Driving Self-*A*wareness Questionnaire* to evaluate participants' awareness of their driving performance is based on widely used measures of self-awareness [e.g. (31–33)]. The questionnaire includes seven questions related to the participants' driving performance, ability to control the vehicle, performing maneuvers, following traffic rules and laws, and navigating successfully to an assigned location. Participants had to answer these questions before (*prediction*) and after (*estimation*) performing the on-road test. Items were scored on a five-point Likert scale, ranging from 1 = very poor to 5 = Excellent. In addition to the participants’ prediction and estimation, the OT and DI also separately graded the same questions following the on-road test, as these items reflect actual driving ability assessed in the evaluation. Thus, four scores were obtained: (1) participants' prediction; (2) participants' estimation; (3) OT rating; and (4) DI rating. After obtaining these four scores, driving self-awareness scores were computed by subtracting the participant's self-estimation of driving performance (i.e., post-on-road test scores) from the DI and OT individual rating scores. This procedure yielded three categories: (1) negative scores, which indicate participant's over-estimation of driving abilities (participant's estimation was higher than DI/OT ratings); (2) positive scores, which indicate under-estimation of driving abilities (DI/OT ratings were higher than participant's estimation); and (3) accurate or close-to-accurate estimation (DI/OT ratings are equal or almost equal to participant's estimation).

*Fitness to drive* decision was based on both off and on-road evaluations (not including the self-awareness score), and it involved a clinical reasoning process done only by the OT. This decision was made by (a) comparing scores from off-road evaluations to norms and/or prior assessments the client has undergone, and (b) calculating scores from the on-road driving evaluation based on observable driving behaviors noted independently by both OT and DI. In short, drivers who are able to manage the vehicle, make appropriate driving decisions, follow the rules of the road, and navigate the car safely during the on-road driving evaluation are classified as fit to drive (34). Based on this information, the OT calculated the driving risks and benefits and made a professional judgment of current and future driving ability.

### Data analysis

Using SPSS software, independent *t*-tests were performed to compare the mean differences in cognitive and motor processing speed and driving self-awareness scores between the two groups (fit and unfit). In addition, a binary logistic regression analysis was performed to examine whether cognitive and motor processing speed or driving self-awareness could predict fitness to drive. For the logistic regression analysis, the driving self-awareness score computed by subtracting the participant's self-estimation of driving performance from the DI rating was used. This score was used to reduce potential bias since the DI was blinded to the in-clinic evaluation results and had no prior knowledge about the participants' abilities.

## Results

### Participants' characteristics

Following a comprehensive evaluation process (as described above), the OT classified participants as fit (*n* = 23) or unfit to drive (*n* = 16). Table 1 summarizes the participants' characteristics by group. Both groups were predominantly male: 87% males in the fit-to-drive group and 75% in the unfit group. Age [*t*(37) = −1.21, *p *= 0.23] and years of education [*t*(32.81) = 0.98, *p *= 0.33] were similar between groups. Among the fit-to-drive group, the most frequent reason for referral to a driving evaluation was a change in physical health or change in health status, followed by cognitive decline and age or necessary evaluation for a driver's license renewal (respectively). Among the unfit-to-drive group, the primary reason for referral was cognitive decline, followed by changes in physical health or health status.

**Table 1:** Description fit to drive and unfit to drive participants characteristics.

	Fit (*n* = 23)		Unfit (*n* = 16)		*p*
	*n*	%	*n*	%	
**Sex (*n*)**					
Male	20	87	12	75	
Female	3	13	4	25	0.41
**Age [mean (SD)]**	71.70 (11.07)		75.56 (7.51)		0.23
**Years of education [mean (SD)]**	15.45 (5.41)		14.08 (2.9)		0.33
**Reason for referral**					
Change in physical health or change in health status	12	52.2	5	31.3	
Age or driver's license renewal	3	13.0	0	0	
Cognitive decline	8	34.8	8	50.0	
Mental health reason (e.g., depression)	0	0	1	6.3	
Family concerns	0	0	1	6.3	
Previous involvement in a car accident	0	0	1	6.3	

### Differences between groups

Independent-sample *t*-tests were conducted to compare mean scores of motor and cognitive processing speed, OT and DI ratings, and driving self-awareness variables between the two groups. These comparisons are described in Table 2. All comparisons were significantly different between the two groups, except for the participants' prediction and estimation of driving (pre- and post-road test).

**Table 2:** Differences between groups in the study's outcome measures.

	Fit (*n* = 23)		Unfit (*n* = 16)		*t*	*p*
	Mean	SD	Mean	SD		
**Cognitive processing speed**						
CTT1 global (seconds)	76.67	43.77	185.84	110.60	−3.54	0.003
**Motor processing speed**						
Physical response time (seconds)	0.70	0.10	0.90	0.27	−2.38	0.033
**Awareness Scores**						
Participant's prediction of driving (pre-road test self-awareness)	30.91	3.86	29.43	5.87	0.410	0.410
Participant's estimation of driving (post-road test self-awareness)	30.04	3.99	27.64	5.76	0.143	0.143
OT rating	30.65	2.53	18.33	4.45	10.89	<0.001
DI rating	29.13	3.75	14.33	3.27	12.51	<0.001
Driving self-awareness 1 (DI-participant's estimation)	−0.91	4.55	−13.00	5.62	7.17	<0.001
Driving self-awareness 2 (OT-participant's estimation)	0.61	3.82	−9.21	5.65	5.76	<0.001

Participants in the fit-to-drive group had shorter cognitive and motor processing speed time compared to the unfit-to-drive group. No significant differences were found between the two groups in participants' prediction of driving (pre-road test) or estimation of driving scores (post-road test). Both the OT and DI rating of the participants' driving abilities was significantly higher for the fit-to-drive group than for the unfit-to-drive group.

The fit-to-drive group had accurate or close-to-accurate driving self-awareness scores, whereas the unfit-to-drive group over-estimated their driving abilities. Similarly, driving self-awareness scores with the OT and DI ratings (Driving self-awareness 1 and 2, i.e., when participants' estimations were subtracted from the DI or OT ratings) were significantly different between the two groups.

### Prediction of fitness to drive

Binary logistic regression for all participants in both groups was performed to examine whether motor and cognitive processing speed and driving self-awareness (DI score relative to participant's estimation) can predict fitness to drive. Analysis showed and explained 96.9% of the variance for fit/unfit to drive. Driving self-awareness was the only significant predictor of fitness to drive (*p *= 0.04) Results of the regression analysis are presented in Table 3.

**Table 3:** Binary logistic regression to predict fitness to drive.

Predictor	*β*	S.E.	Wald	Odds Ration	Confidence interval		Sig.
					Lower	Upper	
Cognitive processing speed	0.02	0.02	1.27	1.02	0.98	1.06	0.26
Motor processing speed	4.75	8.43	0.32	115.59	0.00	1.7	0.57
Self-awareness (DI-participant's estimation)	−0.43	0.21	4.18	0.65	0.43	0.98	0.04
Constant	−8.78	6.25	1.97	0.00			0.16

## Discussion

This study's primary finding is that driving self-awareness significantly predicted participants' fitness to drive above and beyond cognitive and motor processing speed. This finding stresses the importance of addressing self-awareness in driving evaluation with clients at risk for compromised driving skills. The results of this study are in line with previous studies in the field, emphasizing that good driving self-awareness or general self-awareness influence older adults' driving safety and performance (35–38), and persons with traumatic brain injury coping strategies while driving (23, 38).

In this study, self-awareness was assessed in the context of driving using a discrepancy method comparing the OT and DI impressions of the participants' driving with participants' estimation of their actual driving performance. Results indicated that the two groups did not differ in their self-ratings of driving performance. However, the unfit-to-drive group significantly overestimated their performances compared to the assessments made by the DI, while the fit-to-drive group made more accurate self-ratings. Thus, participants in the unfit-to-drive group did not notice and report their driving errors, difficulties, and/or need for help (21, 39). The information provided by driving self-awareness is essential for competency in driving because individuals aware of their errors, difficulties, and limitations while driving can make better decisions and adjustments while driving to ensure independence and safety.

In the current study, both the fit and unfit-to-drive groups perceived their driving performance similarly and as relatively efficient. Even following the actual on-road performance, the unfit-to-drive group did not change their estimation of their driving, and it was similar to that of the fit-to-drive group. Older drivers, regardless of health status, tend to highly rate and overestimate their driving ability (36, 40, 41). Moreover, Kosuge and colleagues (41) report that older drivers who overestimate their performance are likely to drive faster and pay less attention to road signs, supporting a previous study that suggested that inaccurate self-assessments of driving abilities are associated with traffic violations (42). The unfit-to-drive group's self-reporting pattern and their estimations that were similar to those of the fit-to-drive group could be attributed to their inability to notice and acknowledge that their driving performance was below expectations (23). Furthermore, even though not statistically different, their age was older than the fit-to-drive group. Along this line of thought, it has been suggested that increasing the accuracy of one's self-assessment can potentially improve driving performance, including choice of driving speed and scanning behavior (41). Thus, driving safely and efficiently (i.e., adjustment and decision-making during driving) require awareness of how to operate the car and also having traffic insight, and awareness of cognitive abilities. Such skills increase attention and caution during driving (23, 38).

As expected, we found that the fit-to-drive group demonstrated better cognitive and motor processing speed skills compared to the unfit-to-drive group. These findings align with the literature that supports using in-clinic evaluation to provide indicative information regarding the participants' ability to drive and identifying at-risk drivers (7, 29, 43). In addition, as anticipated, both the DI and OT were more impressed by the driving performance of the fit-to-drive group than the unfit-to-drive group. Again, it should be noted that only the OT weighed in on the final decision as to whether a driver was fit or unfit to drive. These findings support the distinction and group allocation made by the OT between the driving abilities of the two groups as perceived; they also support the sensitivity of the driving self-awareness to capture the driving abilities of each group.

The current study has several limitations. First, the sample was heterogeneous regarding diagnosis and reasons for driving evaluation referral. Using a larger sample and dividing participants into subgroups according to diagnosis, for example, may highlight each subgroup's unique characteristics, patterns, and specific needs. Second, this sample was obtained from a practicing clinic, thus limiting data collection. It is suggested that future studies expand the data collection procedure and potentially include participants that might not have resources or access to occupational therapy driving rehabilitation centers. Third, the in-clinic evaluation included a comprehensive assessment battery to evaluate the participants' physical, vision, and cognitive skills. However, some participants did not have scores for all assessments and therefore were not included in the current study. It is recommended that future studies incorporate more cognitive assessments to further characterize the samples. Furthermore, the determination of whether participants were fit or unfit to drive in this study was based on detailed on and off-road evaluation. Some neuropsychological measures used in the off-road evaluation were found to predict fit/unfit to drive with 73% sensitivity and 76% specificity (44). On-road testing is considered the “golden standard” in determining driving fitness. Yet, on-road evaluation reliability and validity are at times questionable, due to methodological and environmental influences (45). Finally, future studies should include more than a single on-road experience to increase the sensitivity to detecting changes in estimation and to obtain a broader understanding of driving self-awareness. Despite the aforementioned limitations, the current study points to the importance of self-awareness in the context of relevant, everyday activities (37, 46, 47), specifically with regard to driving.

In conclusion, this study highlights the importance of a formal evaluation of driving self-awareness in determining fitness to drive among adults referred to an OT evaluation. One could argue that the OT and DI impressions of driving ability could be enough to determine fitness to drive (48). However, the unique information provided by driving self-awareness shows whether clients understand what is required to drive safely and whether they believe they possess these skills. Such information may promote better decision-making and adjustment during driving, resulting in safer driving. Additionally, comparing the OT, DI, and clients' self-evaluations following driving, could be used in driving rehabilitation as a mean to increase self-awareness (21). Practitioners should be aware of the close relationship between driving self-awareness, safe driving performance, and fearing losing one's autonomy if one is no longer able to drive. Following a driving fitness evaluation, practitioners may focus on interventions that address driving self-awareness in order to help to maintain driving competency. The goal is to help the individual continue mobility within the community, especially in light of advancing age and/or the presence of impairments and chronic conditions.

## Data Availability

The raw data supporting the conclusions of this article will be made available by the authors, without undue reservation.
